# Pathogenicity of Highly Pathogenic Avian Influenza A(H5N1) Viruses Isolated from Cats in Mice and Ferrets, South Korea, 2023

**DOI:** 10.3201/eid3010.240583

**Published:** 2024-10

**Authors:** Il-Hwan Kim, Jeong-Hyun Nam, Chi-Kyeong Kim, Yong Jun Choi, Hyeokjin Lee, Bo Min An, Nam-Joo Lee, Hyoseon Jeong, Su-Yeon Lee, Sang-Gu Yeo, Eun-Kyoung Lee, Youn-Jeong Lee, Jee Eun Rhee, Sang Won Lee, Youngmee Jee, Eun-Jin Kim

**Affiliations:** Korea Disease Control and Prevention Agency, Cheongju, South Korea (I.-H. Kim, J.-H. Nam, C.-K. Kim, Y.J. Choi, H. Lee, B.M. An, N.-J. Lee, H. Jeong, S.-Y. Lee, S.-G. Yeo, J.E. Rhee, S.W. Lee, Y. Jee, E.-J. Kim);; Animal and Plant Quarantine Agency, Gimcheon, South Korea (E.-K. Lee, Y.-J. Lee)

**Keywords:** influenza, highly pathogenic avian influenza A virus, H5N1, mammal pathogenicity, mammal-to-mammal transmission, outbreak, cats, mice, ferrets, respiratory infections, viruses, zoonoses, South Korea

## Abstract

The prevalence of highly pathogenic avian influenza (HPAI) A(H5N1) viruses has increased in wild birds and poultry worldwide, and concomitant outbreaks in mammals have occurred. During 2023, outbreaks of HPAI H5N1 virus infections were reported in cats in South Korea. The H5N1 clade 2.3.4.4b viruses isolated from 2 cats harbored mutations in the polymerase basic protein 2 gene encoding single amino acid substitutions E627K or D701N, which are associated with virus adaptation in mammals. Hence, we analyzed the pathogenicity and transmission of the cat-derived H5N1 viruses in other mammals. Both isolates caused fatal infections in mice and ferrets. We observed contact infections between ferrets, confirming the viruses had high pathogenicity and transmission in mammals. Most HPAI H5N1 virus infections in humans have occurred through direct contact with poultry or a contaminated environment. Therefore, One Health surveillance of mammals, wild birds, and poultry is needed to prevent potential zoonotic threats.

Since the emergence of the highly pathogenic avian influenza (HPAI) A(H5N1) virus (A/chicken/Scotland/59) in Scotland, UK, several outbreaks of H5Nx viruses have been reported in poultry worldwide (*1*). In 1996, an HPAI H5N1 virus, A/goose/Guangdong/1/1996 (Gs/GD), was identified, and the Gs/GD lineage H5 viruses have been circulating in poultry and wild aquatic bird reservoirs for >25 years (*1*,*2*). HPAI H5N1 viruses pose a global threat to the poultry industry and public health because of frequent outbreaks in chicken, ducks, and other poultry (*3*). According to the World Health Organization, 882 cases of avian influenza A(H5N1) infections in humans have been reported globally from January 1, 2003, to December 21, 2023, resulting in 461 deaths (52% mortality rate) (*4*).

Since 2005, HPAI H5N1 viruses have diversified genetically, forming numerous genotypes through reassortment with other avian influenza A viruses (*5*). HPAI H5N1 clade 2.3.4.4b viruses of the Gs/GD lineage emerged in Europe in 2020, causing outbreaks in wild birds and poultry in many countries (*5*). The spread of clade 2.3.4.4b viruses was reported in 26 countries worldwide; the virus infected >48 mammal species (*2*,*5*–*7*). In 2022, mass deaths of >20,000 sea lions from HPAI H5N1 infections were confirmed along the coast of South America, including coastal Peru, Chile, Argentina, Uruguay, and Brazil. In addition, in 2023, unusual deaths of cats were reported in Poland (*8*–*10*). Therefore, concerns about the risk for interspecies transmission and human-to-human spread of H5N1 viruses have been growing because of the acquisition of interhost transmission capability and the increase in HPAI H5N1 viruses found in mammals (*11*).

In South Korea, HPAI H5N1 clade 2.3.4.4.b viruses were identified in wild birds in 2021, which was followed by infection outbreaks in poultry farms (*12*). During autumn 2022, introductions of >2 types of HPAI H5N1 clade 2.3.4.4b viruses that originated from Eurasian breeding grounds and North America occurred simultaneously, and various genotypes were subsequently detected in wild birds and domestic poultry (*13*,*14*). During July 2023, unusual deaths of cats at animal shelters occurred in the Yongsan and Gwanak Districts of Seoul, South Korea, caused by HPAI H5N1 viruses (*15*,*16*); viruses isolated from cats were obtained from each animal shelter. We analyzed the pathogenicity and transmission characteristics of 2 cat-derived virus isolates by using molecular methods and by conducting experiments in mouse and ferret infection models. We performed all animal experiments in strict accordance with general animal care guidelines mandated under the Guidelines for Animal Use and Care of the Korea Disease Control and Prevention Agency (KDCA).

## Materials and Methods

### Cells

We grew and maintained MDCK cells (American Type Culture Collection, https://www.atcc.org) in Eagle’s Minimum Essential Medium (WELGENE, https://www.welgene.com) containing 5% fetal bovine serum, 1 mmol/L l-glutamine, and penicillin/streptomycin (Thermo Fisher Scientific, https://www.thermofisher.com). We incubated the cells at 37°C in 5% CO_2_ until use.

### Virus Distribution

The Animal and Plant Quarantine Agency (APQA), South Korea, provided 3 HPAI H5N1 virus isolates and deposited their whole-genome sequences in the GISAID EpiFlu database (http://www.gisaid.org). We propagated the isolates in specific pathogen-free embryonated chicken eggs (second passage) and confirmed that their sequences were identical to those provided by the APQA (Table 1). A/duck/Korea/H493/2022(H5N1) (GISAID accession no. EPI_ISL_15647834) originated from a duck farm in the Yecheon area in October 2022. A/feline/Korea/M302–6/2023(H5N1) (accession no. EPI_ISL_18819809) was from the Yongsan District, and A/feline/Korea/M305–7/2023(H5N1) (accession no. EPI_ISL_18819807) was from the Gwanak District; both of those viruses from cats were obtained from animal shelters during July 2023.

**Table 1:** Characteristics of highly pathogenic avian influenza A(H5N1) viruses isolated from cats, South Korea, 2023*

Virus	Origin	Passage no.†	Clade	MLD_50_‡	Abbreviation
A/duck/Korea/H493/2022	Trachea from duck carcass	2	2.3.4.4b	10^4.8^	YC/2022
A/feline/Korea/M302-6/2023	Trachea from cat carcass	2	2.3.4.4b	10^1.5^	YS/2023
A/feline/Korea/M305-7/2023	Nasal swab sample from cat	2	2.3.4.4b	10^0.5^	GA/2023

*MLD_50_, 50% median lethal dose. †Passage number of virus in embryonated eggs. ‡MLD_50_ was determined by measuring 50% tissue culture infectious dose/mL.

### Genetic and Phylogenetic Analysis

We extracted virus RNA by using the RNeasy Mini Kit (QIAGEN, https://www.qiagen.com) and performed gene amplification and library preparation by using the Illumina Microbial Amplicon Prep-Influenza A/B kit (Illumina, https://www.illumina.com). Subsequently, we sequenced whole genomes of the viruses on a MiSeq instrument by using MiSeq Reagent Kit v2 (Illumina) to obtain 2 × 150-bp read lengths. For phylogenetic analysis, we searched for sequences, other than those analyzed in this study, in the GISAID database. We inferred phylogenetic relationships of sequences obtained in this study by using the maximum-likelihood method, 1,000 bootstrap values, and MEGA 7 software (*17*). 

### Virus Titrations

We determined virus titers of oropharyngeal and cloacal swab samples, nasal washes, and homogenized tissue samples by performing endpoint titrations in MDCK cell monolayers. We inoculated MDCK cells with 10-fold serial dilutions of each sample prepared in fetal bovine serum–free medium containing L-1-tosylamido-2-phenylethyl chloromethyl ketone–treated trypsin and penicillin/streptomycin. After a 72-hour incubation at 37°C, we detected viruses in a standard hemagglutination assay by using 0.5% turkey erythrocytes. We expressed mean virus titers as log_10_ 50% tissue culture infectious dose (TCID_50_). The detection limit was 0.5 log_10_ TCID_50_/mL. We estimated virus titers by using *t*-tests and 2-way analysis of variance in GraphPad Prism 9 (GraphPad Software Inc., https://www.graphpad.com).

### Neuraminidase Inhibitor Resistance

We used the neuraminidase (NA) inhibitors oseltamivir and peramivir (Cayman Chemical, https://www.caymanchem.com) and zanamivir (Sigma-Aldrich, https://www.sigmaaldrich.com) to assess drug susceptibility of the 3 virus isolates. We used a fluorescence assay containing the 2′-(4-methylumbelliferyl)-α-D-*N*-acetylneuraminic acid substrate (Sigma-Aldrich) (*18*,*19*). We normalized influenza viruses to equivalent NA activities and incubated virus samples with 10-fold serial dilutions (0–30,000 nmol/L) of oseltamivir, zanamivir, or peramivir. We measured the fluorescence signal by using a Mithras LB 940 reader (Berthold Technologies, https://www.berthold.com) at excitation/emission wavelengths of 355/460 nm. We estimated the 50% inhibitory concentration (IC_50_) for each sample from dose-response curves by using the sigmoidal, 4-parameter, logistic nonlinear regression equation in GraphPad Prism 9. To assess neuraminidase inhibitor (NAI) resistance, we divided the IC_50_ value of the virus being analyzed by the IC_50_ value of the NAI-sensitive influenza A(H1N1)pdm09 virus strain, which has the amino acid H274 in neuraminidase, making it NAI susceptible.

### Experimental Infections of Mice and Ferrets

We anesthetized groups of 6-week-old BALB/c mice (SAMTAKO, http://www.samtako.com) (n = 5/group) with ketamine and intranasally inoculated them with 50 µL of 10^0^–10^6^ TCID_50_/mL of virus. After virus inoculation, we weighed the mice and monitored them for clinical signs and death for 14 days. For virus replication studies, we intranasally inoculated 15 mice per group with 50 µL of 10^3^ 50% median lethal dose (MLD_50_)/mL. We euthanized 5 mice per group on days 3, 5, and 7 postinoculation and assessed virus titers in brain, trachea, nasal turbinate, lung, heart, liver, kidney, spleen, and intestinal samples.

We anesthetized 20–22-week-old ferrets (IDBio, http://www.idbio.co.kr) (n = 12/group) with ketamine and intranasally inoculated them with 1 mL of 10^3^ MLD_50_/mL of virus. After virus inoculation, we weighed and monitored 3 ferrets per virus group for clinical signs and death for 14 days. We used the remaining 9 ferrets per group for virus replication studies. We euthanized 3 ferrets per virus group on days 3, 5, and 7 postinoculation and assessed virus titers in brain, trachea, nasal turbinate, lung, heart, liver, kidney, spleen, and intestinal samples. To assess virus transmission via contact infection, we intranasally inoculated 1 ferret (per virus) with 1 mL of 10^3^ MLD_50_/mL virus and then housed serologically-naive ferrets (n = 2) in the same cage the next day (1 cage/virus). We collected nasal wash samples from each ferret on days 3, 5, and 7 postinoculation and measured virus titers. We euthanized mice and ferrets showing >20% body weight loss, which we considered a humane endpoint.

### Serologic Tests

We collected blood samples from ferrets in the infection groups 14 days postinfection and in the transmission groups 14 days after contact with a virus-infected ferret. We determined seroconversion by using a microneutralization assay.

## Results

### Genetic Characterization of H5N1 Viruses Isolated from Cats

The Korea Disease Control and Prevention Agency (KDCA) received 2 different HPAI H5N1 viruses from cats in animal shelters that were collected by APQA, which we used to characterize infections in other mammals. We used the 2 H5N1 viruses, A/feline/Korea/M302–6/2023(H5N1) from Yongsan (abbreviated as YS/2023) and A/feline/Korea/M305–7/2023(H5N1) from Gwanak (abbreviated as GA/2023), to determine how the pathogenicity and transmission of those H5N1 viruses differed from previously prevalent H5N1 viruses. We also analyzed the virus isolated from a duck, A/duck/Korea/H493/2022(H5N1) (abbreviated as YC/2022), representing the first poultry outbreak in autumn 2022.

We conducted whole-genome sequence analysis to confirm genetic characteristics of the YC/2022, YS/2023, and GA/2023 viruses and observed polybasic residues (REKRRKR/GLF) within the cleavage sites of hemagglutinin (HA), classifying all 3 viruses as HPAI. Phylogenetic analysis confirmed the viruses belonged to clade 2.3.4.4b (Table 1; Appendix Figure). YS/2023 and GA/2023 viruses shared 99.9%–100% genetic similarity (Appendix Table). They also showed close genetic relatedness to H5N1 viruses that have been circulating in wild birds and poultry in Asia since 2022, including in South Korea, China, and Japan (*15*).

We identified amino acid substitutions related to mammal adaptation in both YS/2023 and GA/2023 viruses. In both viruses, we found mutations S123P, S133A, and T156A (H5 numbering), which enhance binding affinity of the HA protein to α2,6-sialic acid on the host cell surface and contribute to increased mammal receptor tropism (*20*; J. Yang et al., unpub. data, https://doi.org/10.1101/2024.07.09.602706). In addition, in the polymerase basic 2 (PB2) gene segment, we identified mutations encoding D701N in YS/2023 and E627K in GA/2023; both substitutions are mammal-adapting mutations known to increase polymerase activity and virulence in mammals (Table 2) (*21*–*23*). However, mutations associated with antiviral drug resistance, such as H274Y in NA and S31N in the matrix protein, were not detected (*21*,*24*).

**Table 2:** Comparisons of major amino acid substitutions in protein segments from highly pathogenic avian influenza A(H5N1) viruses isolated from cats, South Korea, 2023*

Virus	HA†						PB2‡					NA§		M¶
	123	133	156	222	224		526	591	627	701		274		31
A/duck/Korea/H493/2022	**P**	**A**	**A**	Q	G		K	Q	E	D		H		S
A/feline/Korea/M302-6/2023	**P**	**A**	**A**	Q	G		K	Q	E	**N**		H		S
A/feline/Korea/M305-7/2023	**P**	**A**	**A**	Q	G		K	Q	**K**	D		H		S

*Bold letters indicate substitutions in each virus protein. HA, hemagglutinin; M, matrix protein; NA, neuraminidase; PB2, polymerase basic protein 2. †H5 numbering was used. S123P, S133A, T156A, Q222L, and G224S affect α2,6-sialic acid receptor binding affinity. ‡K526R, Q591K, E627K, and D701N enhance virus replication in mammals. §H274Y affects inhibition of NA. ¶S31N affects matrix protein inhibition.

### Virus Pathogenesis in a Mouse Model

**Figure 1:**
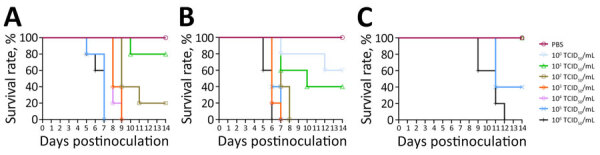
Survival of mice infected with highly pathogenic avian influenza A(H5N1) viruses isolated from cats in South Korea, 2023. Viruses were isolated from 2 cats and 1 duck. A) A/feline/Korea/M302-6/2023; B) A/feline/Korea/M305-7/2023; C) A/duck/Korea/H493/2022. BALB/c mice (n = 5/group) were intranasally inoculated with 10-fold serial dilutions (50 µL of 10° to 10^6^ TCID_50_/mL) of each H5N1 virus. PBS was used as a negative control inoculant. Mice were monitored for 14 days, and survival rates were compared. PBS, phosphate-buffered saline; TCID_50_, 50% tissue culture infectious dose.

We intranasally inoculated 10-fold serial dilutions of infectious dose for each virus into 6-week-old BALB/c mice. After a 2-week observation period, the MLD_50_ values were 10^1.5^ TCID_50_/mL for YS/2023 and 10^0.5^ TCID_50_/mL for GA/2023, whereas the MLD_50_ was 10^4.8^ TCID_50_/mL for YC/2022. The 2 viruses isolated from cats had ≈10-fold difference in MLD_50_ values between them, but their MLD_50_ values were >1,000-fold lower than that of the duck isolate (Table 1; Figure 1).

**Figure 2:**
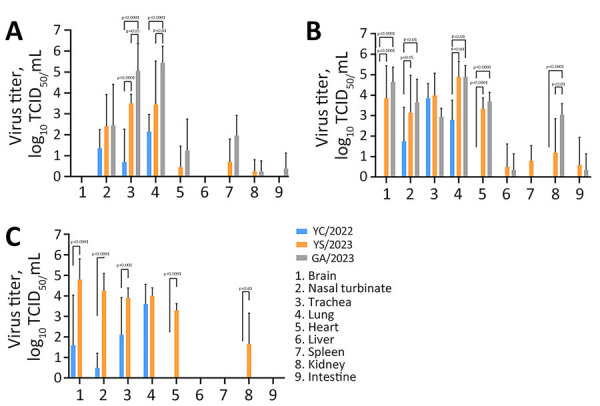
Virus titers in organs of mice infected with highly pathogenic avian influenza A(H5N1) viruses isolated from cats in South Korea, 2023. Viruses were isolated from 2 cats (YS/2023 and GA/2023) and 1 duck (YC/2022). BALB/c mice (n = 15/virus) were inoculated with 50 µL of 10^3^ 50% median lethal dose/mL of each virus; 5 mice/day from each virus group were euthanized on days 3 (A), 5 (B), and 7 (C) postinfection to measure and compare virus titers in organ tissues. GA/2023 virus titers were not measured on day 7 because all of those mice died by day 6 postinfection. p values were calculated by using 2-way analysis of variance. dpi, days postinoculation; GA/2023, A/feline/Korea/M305-7/2023; TCID_50_, 50% tissue culture infectious dose; YC/2022, A/duck/Korea/H493/2022; YS/2023, A/feline/Korea/M302-6/2023.

To assess detailed clinical symptoms and virus replication in internal organs, we intranasally inoculated 50 µL of 10^3^ MLD_50_/mL (YS/2023, 10^4.5^ TCID_50_/mL; GA/2023, 10^3.5^ TCID_50_/mL; YC/2022, 10^7.8^ TCID_50_/mL) of each virus into 6-week-old BALB/c mice (n = 15 in each group). All infected mice exhibited clinical symptoms, such as weight loss, ruffled fur, lethargy, and ataxia, within 5 days postinfection. Virus infection was confirmed in the respiratory tract of all mice on day 3 postinfection, and only viruses isolated from cats were detected in all organs (including the brain) by day 5 postinfection (Figure 2). Furthermore, all virus-infected mice had virus titers in lung, trachea, and nasal turbinate samples beginning on day 3 during the early stage of infection (Figure 2). By day 5, only mice infected with both cat isolates (YS/2023 and GA/2023) had virus titers in 9 organs; we observed high virus titers in brain, nasal turbinate, trachea, lung, and heart samples. In particular, mice infected with GA/2023 exhibited high titers in lung (10^5.4^ TCID_50_/mL) and trachea (10^5.1^ TCID_50_/mL) tissue on day 3 and in lung (10^4.9^ TCID_50_/mL) and brain (10^4.7^ TCID_50_/mL) tissue on day 5, but all mice died before day 7. Mice infected with YS/2023 had high titers (10^3.9^–10^4.9^ TCID_50_/mL) in lung, trachea, and brain tissues on day 5 and in brain (10^4.8^ TCID_50_/mL) and nasal turbinate (10^4.25^ TCID_50_/mL) tissue on day 7. Both cat-derived virus isolates used to infect mice showed a systemic infection pattern and high lethality; high virus titers occurred in most organs, including the brain. The duck isolate, YC/2022, was detected only in the brain and respiratory organs (nasal turbinate, trachea, and lungs) by day 7; virus titers were lower than those for the cat isolates (Figure 2).

### Virus Pathogenesis and Transmission in a Ferret Model

**Figure 3:**
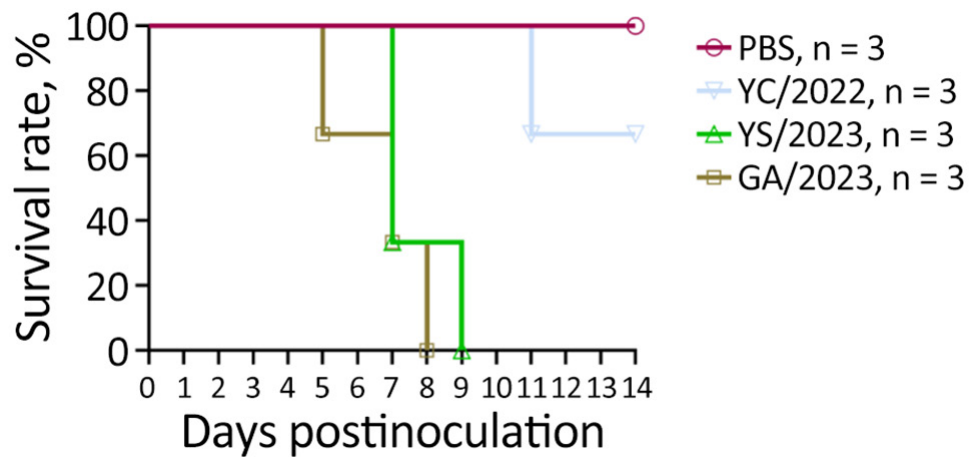
Survival of ferrets infected with highly pathogenic avian influenza A(H5N1) viruses isolated from cats in South Korea, 2023. Viruses were isolated from 1 duck (YC/2022) and 2 cats (YS/2023 and GA/2023). Ferrets (n = 3/group) were intranasally inoculated with 1 mL of 10^3^ 50% median lethal dose of each H5N1 virus; PBS was used as a negative control inoculant. Ferrets were monitored for 14 days, and survival rates were compared. GA/2023, A/feline/Korea/M305-7/2023; PBS, phosphate-buffered saline; YC/2022, A/duck/Korea/H493/2022; YS/2023, A/feline/Korea/M302-6/2023.

**Figure 4:**
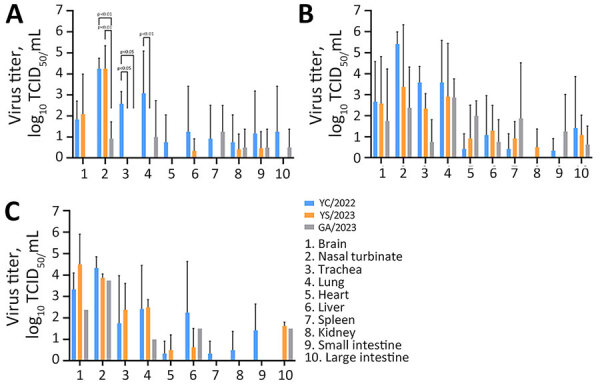
Virus titers in organs of ferrets infected with highly pathogenic avian influenza A(H5N1) viruses isolated from cats in South Korea, 2023. Viruses were isolated from 1 duck (YC/2022) and 2 cats (YS/2023 and GA/2023). Ferrets (n = 9/virus) were inoculated with 1 mL of 10^3^ 50% median lethal dose of each virus, and 3 ferrets/day from each virus group were euthanized on days 3 (A), 5 (B), and 7 (C) postinfection to measure and compare virus titers in organ tissues. p values were calculated by using 2-way analysis of variance. GA/2023, A/feline/Korea/M305-7/2023; TCID_50_, 50% tissue culture infectious dose; YC/2022, A/duck/Korea/H493/2022; YS/2023, A/feline/Korea/M302-6/2023.

Ferrets intranasally infected with the 2 cat-derived H5N1 virus isolates showed severe clinical symptoms, including sneezing, nasal discharge, diarrhea, and neurologic complications. They also exhibited a mean peak reduction in bodyweight of 5.5%–25.7% and fever of 0.7°C–2.6°C above baseline temperature. All ferrets died by day 8 in the GA/2023 infection group and by day 9 in the YS/2023 group (100% mortality rate) (Figure 3). The ferrets infected with the GA/2023 and YS/2023 viruses showed a systemic infection pattern. In ferrets infected with GA/2023, the virus was detected in all organs except the kidneys by day 5, and virus titers of 10^0.8^–10^2.9^ TCID_50_/mL were detected in the brain and respiratory organs. Those titers were lower than titers observed in ferrets infected with YS/2023 (10^2.3^–10^3.4^ TCID_50_/mL) that survived longer (Figure 4). The ferrets infected with YC/2022 showed the highest virus titers in the respiratory tract (10^2.3^–10^5.4^ TCID_50_/mL) until day 5, and infection was observed in all organs. However, 1 ferret died in the YC/2022 group on day 11, resulting in a survival rate of 66.6% (Figure 3).

**Figure 5:**
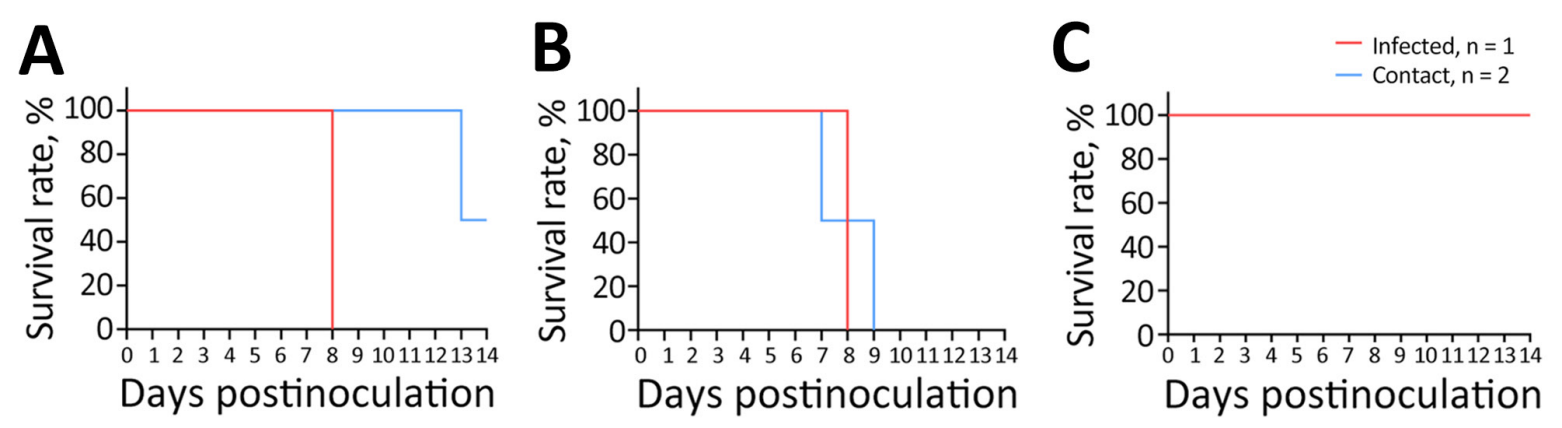
Survival rates after ferret-to-ferret contact transmission in study of pathogenicity of highly pathogenic avian influenza A(H5N1) viruses isolated from cats, South Korea, 2023. Viruses were isolated from 2 cats and 1 duck. A) A/feline/Korea/M302-6/2023; B) A/feline/Korea/M305-7/2023; C) A/duck/Korea/H493/2022. We intranasally inoculated 1 ferret with 1 mL of 10^3^ 50% median lethal dose of each virus (1 ferret/virus) and then housed serologically naive ferrets (n = 2) in the same cage the next day (1 cage/virus). Survival rates for the inoculated and naive ferrets were measured.

**Figure 6:**
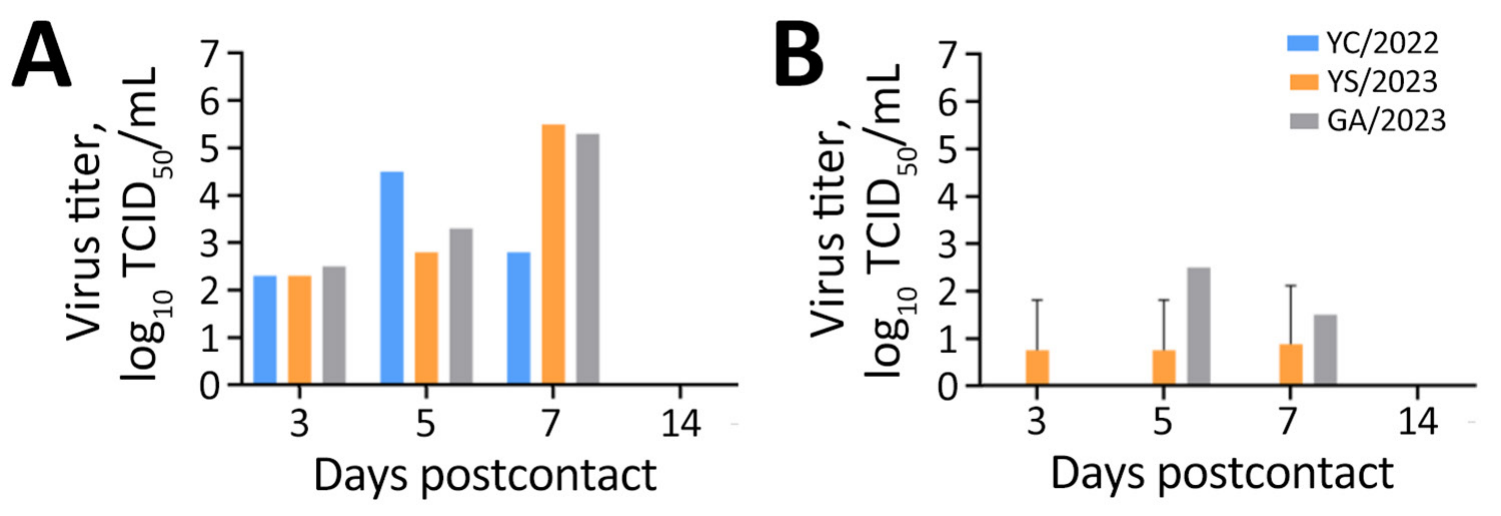
Virus titers after ferret-to-ferret contact transmission in study of pathogenicity of highly pathogenic avian influenza A(H5N1) viruses isolated from cats, South Korea, 2023. Virus titers were measured in nasal washes from ferrets initially inoculated with virus (A) and naive ferrets exposed to the infected ferret (B). Viruses were isolated from 2 cats (YS/2023 and GA/2023) and 1 duck (YC/2022). We intranasally inoculated 1 ferret with 1 mL of 10^3^ 50% median lethal dose of each virus (1 ferret/virus) and then housed serologically naive ferrets (n = 2) in the same cage the next day (1 cage/virus). To evaluate transmission of virus to naive animals, nasal wash samples were collected over time, and virus titers were measured. GA/2023, A/feline/Korea/M305-7/2023; TCID_50_, 50% tissue culture infectious dose; YC/2022, A/duck/Korea/H493/2022; YS/2023, A/feline/Korea/M302-6/2023.

To assess virus transmission via contact with YS/2023, GA/2023, and YC/2022, each virus was intranasally inoculated into 1 ferret and 2 serologically naive ferrets were moved into the same cage as the infected animal. The ferrets inoculated with either cat-derived virus died on day 8 postinoculation. In contrast, only 1 of 2 ferrets in contact with the YS/2023-infected ferret died on day 13; no seroconversion was observed in the surviving ferret. The 2 ferrets in contact with the GA/2023-infected ferret died by day 9 (100% mortality rate) (Figure 5). Virus concentrations increased (10^2.3^–10^5.5^ TCID_50_/mL) in nasal wash samples collected from ferrets exposed to the cat-derived viruses on days 3, 5, and 7, confirming transmission and infection in naive ferrets through contact with infected animals (Figure 6). In contrast, the ferret infected intranasally with YC/2022 did not die, although the virus was detected in nasal washes. Both ferrets in that contact group survived; no virus was detected in nasal washes and no seroconversion was observed, inferring that contact transmission of YC/2022 did not occur.

### Antiviral Drug Susceptibility of Influenza A(H5N1) Viruses Isolated from Cats

We experimentally analyzed NAI susceptibility to evaluate the effectiveness of existing influenza antiviral drugs against YS/2023, GA/2023, and YC/2022 viruses. We compared the IC_50_ values of the 3 viruses with that of an antiviral drug–susceptible human influenza A(H1N1)pdm09 reference virus. The high sensitivity of YS/2023, GA/2023, and YC/2022 viruses to NAIs confirmed the effectiveness of specific antiviral drugs (Table 3).

**Table 3:** Susceptibility of highly pathogenic avian influenza A(H5N1) viruses isolated from cats in South Korea, 2023, to antiviral drugs*

Virus	Subtype	Neuraminidase inhibitors							
		Oseltamivir			Zanamivir			Peramivir	
		IC_50_, nM	Fold change		IC_50_, nM	Fold change		IC_50_, nM	Fold change
A/duck/Korea/H493/2022	H5N1	0.16	0.11		0.19	0.18		0.44	0.775
A/feline/Korea/M302-6/2023	H5N1	0.35	0.24		0.18	0.17		0.2	0.3
A/feline/Korea/M305–/2023	H5N1	0.23	0.15		0.16	0.15		0.2	0.36
Influenza A(H1N1)pdm09	H1N1	1.47	1		1.09	1		0.57	1

*H5N1 virus from 1 duck was compared with 2 viruses isolated from cats. Fold change in drug susceptibility was measured relative to that of influenza A(H1N1)pdm09 containing the wild-type H274 amino acid in neuraminidase. IC_50_, 50% inhibitory concentration.

## Discussion

HPAI H5N1 outbreaks continue worldwide, posing considerable threats to humans and animals. HPAI H5N1 clade 2.3.4.4b viruses have been detected in wild birds and domestic poultry in South Korea (*14*). In addition, infection outbreaks in cats caused by HPAI H5N1 clade 2.3.4.4b viruses occurred in 2 animal shelters in South Korea during July 2023. Both H5N1 viruses isolated from cats had genetic constellations similar to that of the predominant influenza virus circulating in wild birds and poultry in South Korea during 2022–2023. An investigation of the source of infection found that the cats were infected by ingesting raw duck feed contaminated with the prevalent circulating virus. The raw feed–derived viruses were genetically identical to the poultry virus; however, the APQA found 2 mutations related to mammal adaptation (E627K and D701N) in PB2 of the isolates from cats (Y.M. Kang et al., unpub. data). Therefore, it is critical to prevent HPAI virus infections in mammals because avian-derived influenza viruses have been found to mutate after infecting mammals. We performed genetic analysis and animal model experiments to assess the potential mammal-to-mammal transmission and pathogenicity of HPAI H5N1 clade 2.3.4.4b viruses isolated from cat outbreaks in other mammals.

We analyzed 5 amino acids encoded by the HA gene segment (S123P, S133A, T156A, Q222L, and G224S). S123P, S133A, and T156A have been reported to increase mammal receptor affinity by enhancing binding to α2,6-sialic acid (*21*); however, S123P increased the affinity for α2,6-sialic acid only in the presence of E75K, N193K, or R437K substitutions (*20*). YC/2022, YS/2023, and GA/2023 viruses did not have E75K, N193K, or R437K substitutions. Clade 2.3.4.4b viruses have not shown increased affinity for α2,6-sialic acid, even with S133A and T156A substitutions in HA (J. Yang et al., unpub. data). We did not find the Q222L and G224S substitutions, which are associated with strong affinity for α2,6-sialic acid, in YC/2022, YS/2023, and GA/2023 viruses (*20*,*25*). Consequently, we do not consider the effects of HA mutations to be substantial for those viruses.

The H5N1 PB2 substitution E627K was observed in cats in Poland, and the PB2 D701N substitution was observed in sea lions in Argentina (*10*,*26*). According to sequence data registered in GISAID, >50% of human HPAI virus isolates exhibit E627K or D701N substitutions in PB2. Those mammal-adaptive mutations are critical factors that increase replication and virulence of H5N1 viruses in cell culture and animal experiments (*27*–*30*).

Ferrets are useful animal models to study influenza virus transmission and are frequently used for influenza pathogenicity evaluation because they exhibit influenza-like symptoms after infection, including fever, malaise, anorexia, sneezing, and nasal discharge (*31*–*33*). The H5N1 viruses isolated from cats exhibited high virus replication levels and systemic infection along with severe symptoms and high mortality rates in mice and ferrets; in addition, contact transmission among ferrets was confirmed. Therefore, it was inferred that YS/2023 and GA/2023 are highly pathogenic in mammals and are capable of mammal-to-mammal transmission. It was also presumed that the amino acid substitutions E627K and D701N in PB2, previously associated with increased replication and virulence in mammals (*29*), might be responsible for the pathogenicity and transmission of H5N1 viruses in mammals. YS/2023 and the GA/2023 are genetically similar, except for the PB2 substitutions D701N in YS/2023 and E627K in GA/2023. The GA/2023 virus with the E627K substitution showed stronger contact transmission in ferrets than the YS/2023 virus with the D701N substitution. It has been reported that the E627K substitution in PB2 of H5N1 viruses affects airborne transmission in ferrets (*34*). Therefore, the E627K mutation might have a greater effect on transmission of the cat-derived viruses than D701N, although this possibility requires further investigation.

We compared the 2 cat-derived viruses with a duck-derived virus that occupied the same clade as the H5N1 virus circulating in poultry during 2022. However, the first limitation of our study is that direct comparisons of pathogenicity between isolates could not be completely assessed because of virus gene segmental differences. Nevertheless, it was clear that the H5N1 viruses isolated from cats were more pathogenic and transmissible among mammals than the duck-derived virus. Second, we analyzed mouse infections and contact transmission in ferrets, but we did not include an aerosol droplet transmission experiment to analyze the potential for human-to-human transmission. Consequently, assessing the public health risk to humans was also limited.

Increasing transmission of H5N1 viruses among mammals has been observed in countries in South America and in the United States. Most cases of spillover into humans have involved direct contact with infected poultry or a contaminated environment. The risk for human infection from recent outbreaks of HPAI influenza viruses in mammals, including tigers, leopards, domestic cats, domestic dogs, sea lions, and seals, has been assessed as low by the World Health Organization and other experts because of the lack of evidence for human-specific adaptive changes (*6*,*8*,*11*).

In conclusion, the increased pathogenicity and transmission among mammals observed in ferrets exposed to cat-derived HPAI H5N1 viruses indicate a need to conduct surveillance for H5N1 viruses in wild birds and mammals to prepare for potential zoonotic threats. A One Health surveillance approach is crucial, and sharing and integrating information, such as sequencing data, reference viruses, and experimental data, during outbreaks in birds and mammals are essential to prevent human HPAI H5N1 virus infections.

Appendix Additional information for pathogenicity of highly pathogenic avian influenza A(H5N1) viruses isolated from cats in mice and ferrets, South Korea, 2023.
