# Supplementary material for: Pathogenicity of Highly Pathogenic Avian Influenza A(H5N1) Viruses Isolated from Cats in Mice and Ferrets, South Korea, 2023
Source: Emerg Infect Dis. 2024 Oct;30(10):2033–41. doi: 10.3201/eid3010.240583 (PMC11431923; doi:10.3201/eid3010.240583)
Supplement: Appendix — Additional information for pathogenicity of highly pathogenic avian influenza A(H5N1) viruses isolated from cats in mice and ferrets, South Korea, 2023. [file 24-0583-Techapp-s1.pdf]

Article DOI: https://doi.org/10.3201/eid3010.240583

EID cannot ensure accessibility for supplementary materials supplied by authors. Readers who have difficulty accessing supplementary content should contact the authors for assistance.

Pathogenicity of Highly Pathogenic Avian Influenza A(H5N1) Viruses Isolated from Cats in Mice and Ferrets, South Korea, 2023

Appendix

Appendix Table. Nucleotide sequence similarity among gene segments from highly pathogenic avian influenza A(H5N1) viruses (YC/2022, YS/2023, and GA/2023), South Korea*

Virus	HA	NA	M	PB2	PB1	PA	NP	NS
YC/2022	99%	98.7%	99.4%	92.5%	94.2%	94.9%	93.5%	90.8%
YS/2023†	100%	100%	100%	100%	100%	100%	100%	100%
GA/2023	99.9%	99.9%	100%	99.9%	99.9%	100%	99.9%	100%

*YS/2023 and GA/2023 isolates are from cats and YC/2022 isolate is from a duck. HA, hemagglutinin; M, matrix protein; NA, neuraminidase; NP, nucleoprotein; NS, nonstructural protein; PA, polymerase acidic protein; PB1, polymerase basic protein 1; PB2, polymerase basic protein 2.
†Reference virus for nucleotide sequences.

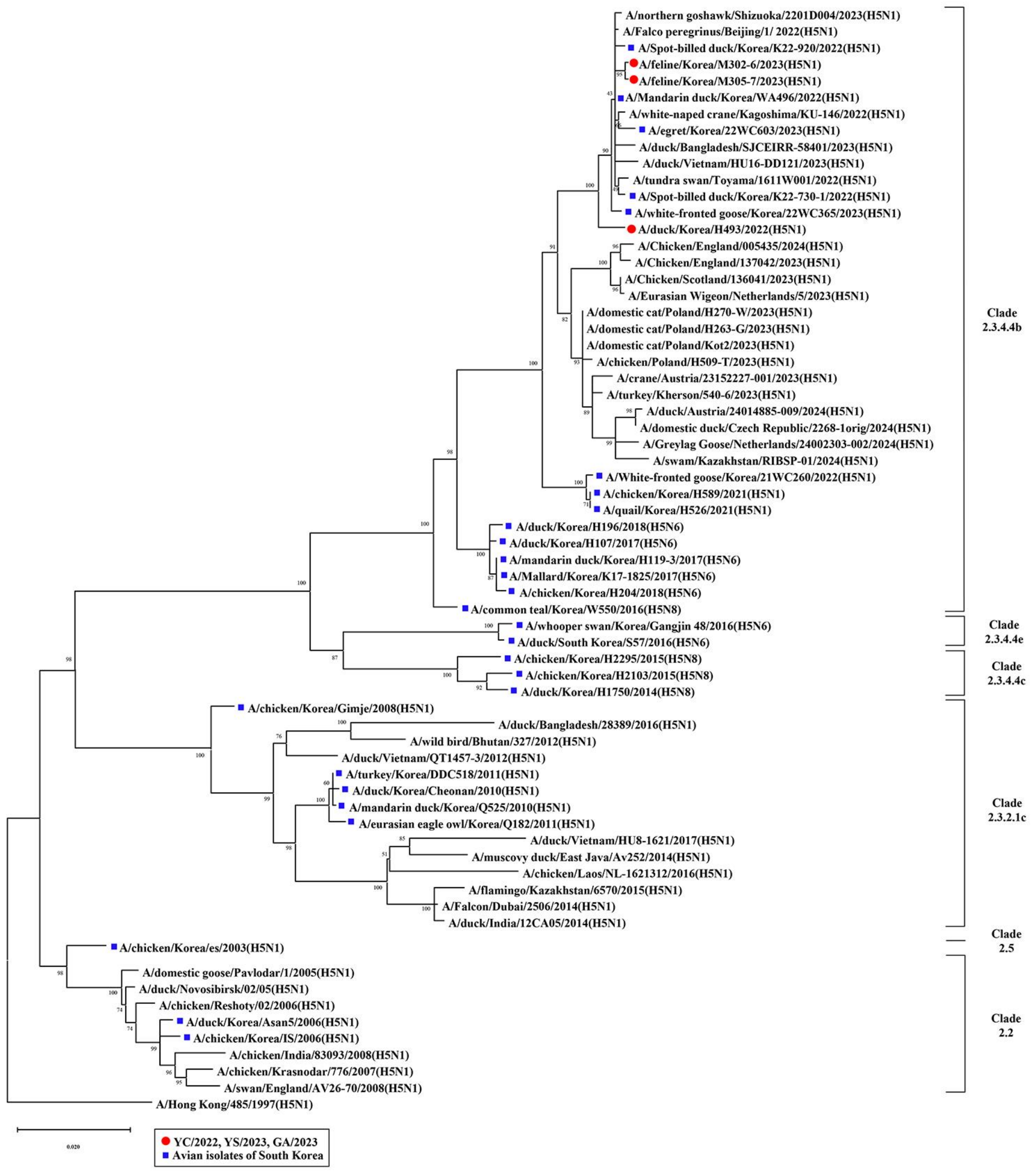

Appendix Figure. Phylogenetic analysis of hemagglutinin gene from highly pathogenic avian influenza A(H5N1) viruses isolated from cats, South Korea. The phylogenetic tree was constructed by using the nucleotide sequences of the hemagglutinin gene segments from YS/2023 and GA/2023 isolates from cats and YC/2022 isolate from a duck together with those of selected influenza A viruses available in GISAID (https://www.gisaid.org). Tree was generated by using the maximum-likelihood method and analyzed by using 1,000 bootstrap replicates. Red circles indicate YC/2022, YS/2023, and GA/2023 from this study; blue squares indicate isolates from South Korea. Scale bar indicates nucleotide substitutions per site.
